# If-then planning, self-control, and boredom as predictors of adherence to social distancing guidelines: Evidence from a two-wave longitudinal study with a behavioral intervention

**DOI:** 10.1007/s12144-021-02106-7

**Published:** 2021-08-14

**Authors:** Maik Bieleke, Corinna S. Martarelli, Wanja Wolff

**Affiliations:** 1 Department of Sport Science, University of Konstanz, Konstanz, Germany; 2 Faculty of Psychology, UniDistance Suisse, Brig, Switzerland; 3 Department of Educational Psychology, University of Bern, Bern, Switzerland

**Keywords:** COVID-19, social distancing, if-then planning (implementation intentions), self-control, boredom

## Abstract

In the wake of the Coronavirus Disease 2019 (COVID-19), social distancing is instrumental for containing the pandemic. To maximize its effectiveness, it is paramount to investigate psychological factors that predict adherence to social distancing guidelines and examine corresponding interventions. We focused on individual differences in if-then planning, self-control, and boredom, and tested an intervention based on if-then planning. We conducted a two-wave longitudinal study combining observational and experimental methods. Participants (*N* = 574, 35.7% female, age: *M* = 37.5 years, *SD* = 10.8) reported their adherence to social distancing guidelines and the perceived difficulty of adherence at T1, along with trait measures of if-then planning, self-control, and boredom. Afterwards, they were randomly assigned to an if-then planning intervention to increase adherence, or to a control intervention. One week later at T2, participants again reported their adherence and the perceived difficulty of adhering. Multiple regression and structural equation modeling were used to establish whether trait if-then planning, self-control, and boredom predicted adherence, and to examine the effects of the if-then planning intervention. Trait if-then planning, self-control, and boredom were associated with T1 adherence, while only if-then planning and boredom predicted T2 adherence. No overall treatment effect of the if-then planning intervention emerged; however, participants who complied with the intervention (75.6%) maintained higher levels of adherence over time than control participants. In sum, individual differences in if-then planning, self-control, and boredom predicted adherence to social distancing guidelines. If-then planning interventions are promising but require further steps to ascertain compliance.

Since its detection in 2019 the coronavirus disease (COVID-19) has spread over almost the entire globe and has been declared a pandemic (WHO, 2020). The rapid and exponential spread of the virus has caused substantial economic and social disruptions that have affected people’s lives and well-being. There is an urgent need for targeted action to help tackle COVID-19. Despite remarkable work in bio-medical and clinical fields, our knowledge about the virus is still limited. Meanwhile, many countries have been employing social distancing guidelines, including reducing physical contact, avoiding social gatherings, and staying at home.

Mathematical modelling of the COVID-19 transmission shows that social distancing measures play a crucial role in reducing the spread of the virus and thus in protecting healthcare systems from being overwhelmed. For example, Kucharski et al. (2020) showed a decline from 2.35 to 1.05 of the viral reproduction number, one week after travel restrictions were introduced in Wuhan in January 2020. However, social distancing measures are effective only if the general public adheres to them. Adherence is generally at a reassuringly high level (Wolff et al., 2020); however, social distancing measures require the highest possible level of adherence and, therefore, even small deviations can pose a threat to their efficacy. Moreover, there is a risk of reduced public acceptance if measures are in place for a prolonged duration (Martarelli & Wolff, 2020)—with some authors estimating that prolonged or intermittent measures might be necessary up until 2022 (Kissler et al., 2020). Thus, our main goal was to elucidate psychological variables that explain variation in adherence to social distancing guidelines and use this framework to test a cost-effective intervention that might facilitate adherence.

Recent work has focused on the negative impact of social distancing, such as lack of freedom, loss of routines, confusion, inadequate supplies, insufficient information, and financial loss, which in turn can cause emotions of fear, anger, anxiety, and boredom (e.g., Barari et al., 2020; Brooks et al., 2020; Park & Park, 2020). Here, we draw upon recent functional theorizing on boredom to illustrate the difficulty some individuals might face in complying with social distancing guidelines. Boredom is associated with negative outcomes like depression (e.g., Goldberg et al., 2011), substance use (Lee et al., 2007), or youth suicide (Heled & Read, 2005). However, recent theorizing highlights that boredom is not negative or positive per se but plays a key role as a driver of behavioral change to seek out more rewarding alternatives (Bench & Lench, 2019; Danckert, 2019; Gomez-Ramirez & Costa, 2017; Wolff & Martarelli, 2020). Adhering to social distancing guidelines comes with reduced behavioral alternatives and thus leads to the experience of boredom (in boredom prone individuals), making adherence more difficult for them. First evidence for the proposed mechanism has been provided by Wolff et al. (2020) who showed that the effect boredom has on adherence to social distancing guidelines is mediated by its perceived difficulty.

In order to adhere to the social distancing guidelines, although boredom signals that one should rather do something else, individuals need to apply self-control. Thus, self-control is a second psychological factor that might be of crucial relevance to allow individuals to adhere to social distancing guidelines. Self-control refers to the ability to volitionally override a default response (e.g., a habitual response) in order to reach a goal (Shenhav et al., 2013). Crucially, the experience of self-control exertion is effortful and aversive (Wolff et al., 2021a) and functional models of self-control suggest that the sensation of effort signals the costs of control (Kurzban et al., 2013; Shenhav et al., 2017). Consequently, self-control is sustained only if the benefits of investing further control outweigh the costs of control. Adhering to the social distancing guidelines, although it is difficult, relies on self-control. Recent empirical work showed that individuals with high trait self-control adhered to the guidelines even though they perceived them as difficult (Wolff et al., 2020).

Taken together, theoretical and empirical work indicates that boredom makes adherence to social distancing guidelines difficult, which in turn lead to less adherence (mediated effect of boredom on adherence), while self-control helps to deal with these difficulties and reduces the negative effect of perceived difficulty on adherence (self-control moderates the effect of difficulty on adherence). This implies that boredom and self-control should be strongly and negatively associated. Indeed, empirical research has observed such a substantial negative correlation between both constructs (e.g., *r* = −.74; Wolff et al., 2021b). Still, boredom and self-control are assumed to make independent contributions to behavior (Bieleke & Wolff, 2021; Wolff & Martarelli, 2020), and in support of this assumption it has been found that self-control and boredom predict health behavior independently from each other (Wolff et al., 2021a). Accordingly, it is reasonable to assume that low self-control and high boredom proneness independently contribute to low levels of adherence as well.

To further increase adherence and to aid people with high boredom proneness and low trait self-control, it seems promising to examine specific self-control strategies. For instance, it has been shown that having strategies specifying how to deal with the pandemic (e.g., searching for ways to make the situation more interesting) is associated with lower boredom and higher satisfaction in comparison to having no strategies (Waterschoot et al., 2021). Making if-then plans represents such a strategy and helps people attain their goals across various domains (Gollwitzer, 2014). When making if-then plans, people mentally link critical situations (e.g., obstacles) and goal-directed behaviors (e.g., ways of dealing with obstacles) in an if-then format: *if (situation), then (behavior)*. This link automates the performance of planned behaviors (Webb & Sheeran, 2007), allowing people to deal swiftly with critical situations. One desirable consequence of if-then planning is that goal striving becomes less demanding, which facilitates the attainment of difficult goals (Freydefont et al., 2016; Legrand et al., 2017). For instance, making if-then plans is associated with reduced activity in brain areas linked to effortful control processes (Wolff et al., 2018). It is thus reasonable to assume that if-then planning helps people adhere to social distancing guidelines, in particular those who find adherence difficult. People vary in their inclination to use if-then plans as a self-control strategy, and a stronger inclination has been associated with better goal attainment (Bieleke & Keller, 2021; Wolff et al., 2021b). Moreover, if-then planning can be conveyed as a self-control strategy (Gollwitzer & Sheeran, 2006).

In sum, the goal of this study was twofold. First, we aimed to replicate and extend recent findings regarding predictors of adherence to social distancing guidelines (self-control and boredom; Wolff et al., 2020). Based on the results of this study, we expected higher trait boredom to be associated with lower adherence to social distancing guidelines via increased perceived difficulty of adherence (i.e., a mediated effect). Specifically, people high in trait boredom should experience boredom more frequently and more intensely than people low in trait boredom (Tam et al., 2021), which should render adherence more difficult and, therefore, less likely. Moreover, we expected high trait self-control to be directly associated with higher adherence, especially among individuals who perceived adherence as difficult (i.e., a moderated effect). Going beyond these replications, we assumed analogous direct and moderating effects with respect to individual differences in the specific self-control control strategy of if-then planning. A further novel contribution is the investigation of the proposed associations in a longitudinal design with a one-week follow up. Figure 1 summarizes the proposed model of the assumed predictors of adherence to social distancing guidelines.

**Fig. 1:**
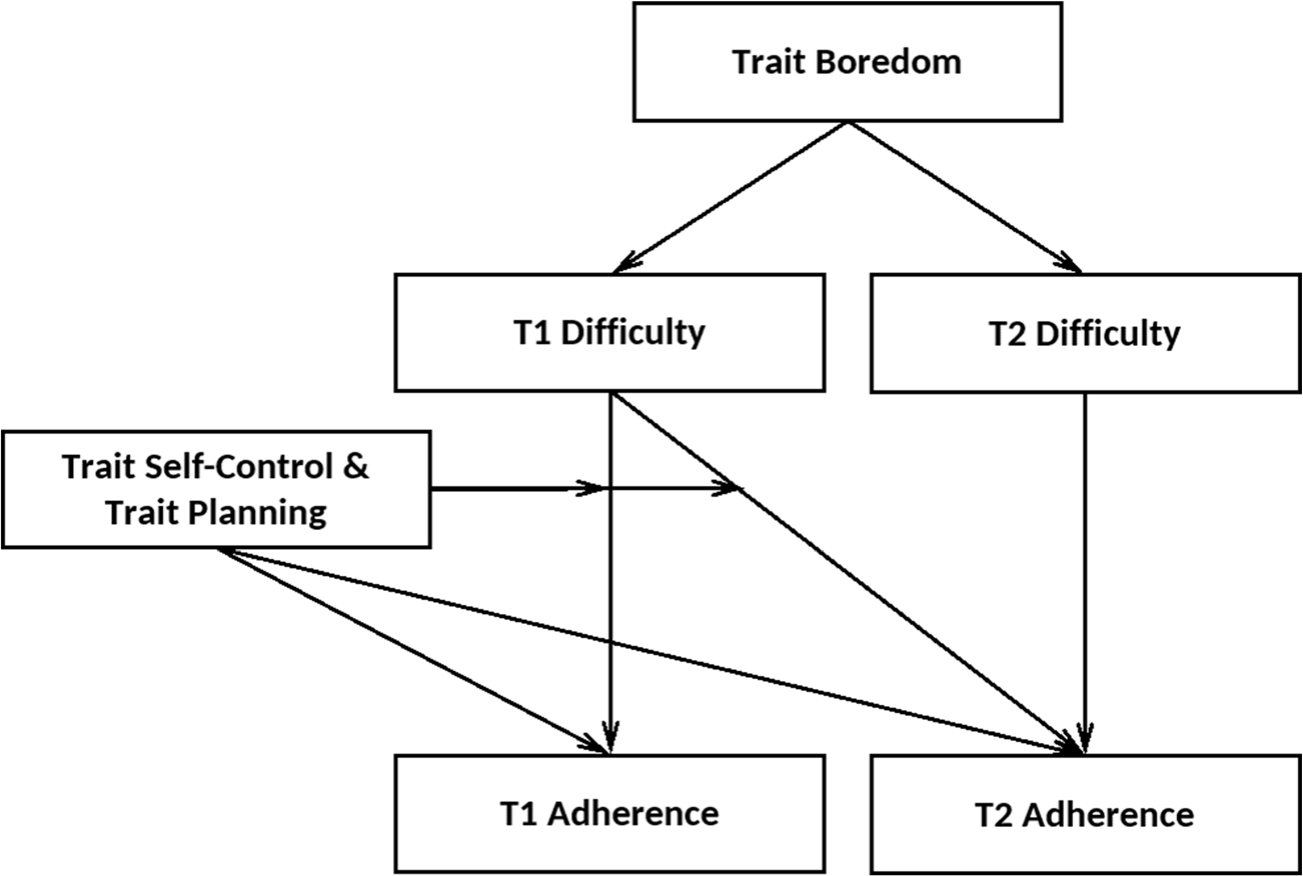
*Proposed Associations of Trait If-Then Planning, Self-Control, and Boredom with the Adherence to Social Distancing Guidelines*. Trait boredom is assumed to decrease adherence by making adherence more difficult (i.e., a mediated effect) without directly affecting adherence. In contrast, trait self-control and trait if-then planning are assumed to increase adherence directly and to mitigate negative effects of difficulty on adherence (i.e., moderated effects). These ideas reflect current theorizing on the role of boredom and self-control for goal pursuit (Wolff & Martarelli, 2020) and are directly derived from empirical research focusing on social distancing (Wolff et al., 2020). The figure shows relationships between trait variables; because the if-the planning intervention could affect difficulty and/or adherence at T2, we tested these relationships while statistically accounting for potential intervention effects

Second, we examined whether an if-then planning intervention increases adherence to social distancing guidelines. Behavioral interventions based on if-then planning are successfully used in various domains of life (e.g., to change health behavior; Adriaanse et al., 2011; Hagger & Luszczynska, 2014) and commonly very effective (average effect size of *d* = .54 across meta-analyses; Keller et al., 2020). Therefore, we reasoned that instructing people to make if-then plans to deal with the difficulties of adhering to social distancing guidelines should facilitate adherence. Previous research indicates that if-then planning requires sufficient commitment to perform the planned behavior (e.g., Achtziger et al., 2012). Moreover, it is conceivable that an if-then planning intervention is particularly helpful for people who have a low propensity to make if-then plans, as well as for people with low self-control or high boredom proneness. Accordingly, we examined whether any intervention effects were moderated by the commitment to adhere to the guidelines or by individual differences in if-then planning, self-control, and boredom.

## Methods

### Participants

We collected data for Time-Point 1 (T1) on 22 and 23 April 2020 from Amazon’s website Mechanical Turk (MTurk; requirements: ≥ 50 HITs, approval rate ≥ 90%, US citizenship). Data collection for Time-Point 2 (T2) started one week later on 30 April and lasted until 4 May 2020. All US states were represented in our dataset except Alaska and North Dakota. Also, larger states were represented by more participants, with most participants (17.6%) coming from California followed by Texas (10.63%), the two states with the highest and second-highest resident populations in the US, respectively. At T1, the 7-day average number of new cases in the US was about 28,500 per day and the 7-day average number of new reported deaths was about 2,100 per day. At T2, the 7-day average number of new cases was between 27,500 and 29,000 per day and the number of new reported deaths was between 1,800 and 1,900 per day (New York Times, 2020). The social distancing guidelines at this time specifically asked to stay at least 6 ft from other people, to not gather in groups, to stay out of crowded places, and to avoid mass gatherings (Centers for Disease Control and Prevention, 2020a).

Simulation studies suggest that at least 250 participants are required to obtain stable correlation estimates (Schönbrodt & Perugini, 2013). We doubled this sample size to 500 to allow for sufficiently powered analyses within the two conditions and to achieve sufficient power (90%) to detect small differences (*d* = 0.2) between them (two-tailed, alpha = .05). Attrition rates in MTurk panel studies are around 20% (Stoycheff, 2016), against which we hedged by further increasing our target sample size to 600. Accordingly, we obtained data from 599 participants in T1, of which 479 participants returned in T2 (i.e., 20.0% attrition). Participants received $2.00 for completing the study (of which $1.00 was paid after T1). In both parts, three datasets with duplicate IP addresses were removed. Data from 22 participants were removed because they failed an instructional manipulation check (Oppenheimer et al., 2009). Accordingly, 574 participants contributed data to T1 and 451 participants contributed data to both T1 and T2.

Participants in our T1 sample (35.7% female, 63.6% male, 0.7% missing/other) were on average 37.5 years old (*SD* = 10.8). The majority reported 13 years or more of education (86.7%) and was either working full-time (61.2%) or self-employed (16.9%). Most participants (62.5%) reported an annual income between $20,000 and $79,999, 24.4% reported to earn ≤ $20,000, and 13.1% reported an income of ≥ $80,000. The study was carried out in accordance with the Declaration of Helsinki and ethical guidelines of the German Psychological Society (DGPs) and the American Psychological Association (APA). All participants provided written informed consent.

### Procedure

Questionnaires were administered online. The full questionnaire is available on OSF (https://osf.io/y2rdk). We used 5-point Likert scales unless otherwise stated (1 = *strongly disagree*, 2 = *somewhat disagree*, 3 = *neither agree nor disagree*, 4 = *somewhat agree*, 5 = *strongly agree*).

#### Measurements and Intervention at T1

At the beginning of T1, participants gave their informed consent and confirmed to be at least 21 years of age, followed by an instructional manipulation check. Next, we provided a definition of social distancing according to guidelines by the US government and assessed *adherence to social distancing measures* with one item (“I stick to the social distancing guidelines”) and the *difficulty of adhering to social distancing measures* with 5 items (“It is difficult for me to stick to the social distancing guidelines”, “I need willpower to adhere to the social distancing guidelines”, “Adhering to the social distancing guidelines bores me”, “Boredom makes it difficult to follow the social distancing guidelines”, “I need my willpower to avoid breaking the social distancing guidelines out of boredom”). The answers to these items were given on 5-point Likert scales (1 = *strongly disagree*, 5 = *strongly agree*) and answers on the difficulty items were averaged into a single score. This way of measuring adherence to social distancing and its difficulty with self-reports has already been used in previous research (with α = 0.87 for the difficulty scale; Wolff et al., 2020) and validated in the context of the COVID-19 pandemic (e.g., homeschooling; Martarelli et al., 2021). Moreover, self-report measures are well-suited for capturing actual social distancing behavior (Gollwitzer et al., 2020). Thus, while there are certainly limits of assessing complex behaviors with self-report (e.g., memory biases), the method is established in research on social distancing and allows to collect data from many individuals quickly, easily, and at low costs.

Afterwards, participants worked on the 8-item *If-Then Planning Scale* (ITPS; sample item: “I think about when and where decisive moments for the achievement of my goals could occur”; Bieleke & Keller, 2021), the 8-item *Short Boredom Proneness Scale* (SBPS; sample item: “I often find myself at ‘loose ends,’ not knowing what to do”; Struk et al., 2017), and the 20-item *Capacity for Self-Control Scale* (CFSCS; sample item: “I am able to resist temptations”; Hoyle & Davisson, 2016). These scales capture individual differences in if-then planning, boredom, and self-control, respectively. We used 5-point Likert scales for all of these scales (1 = *strongly disagree*, 5 = *strongly agree*). Previous research showed that the ITPS and the CFSCS (α = 0.85 and 0.92, respectively, Bieleke & Keller, 2021) as well as the SBPS (α = 0.88; Struk et al., 2017) are reliable instruments. The scores on each of the three scales were averaged.

Participants were then randomly assigned to one of two conditions. In the *control condition*, we presented ten situations recommended by the US Centers for Disease Control and Prevention as key times to wash one’s hands to stay healthy (e.g., “Before, during, and after preparing food”; Centers for Disease Control and Prevention, 2020b). Participants typed each of these key times into text boxes. In the *planning condition*, participants were instructed to set the goal “I will adhere to the social distancing guidelines” and to type it into a textbox. Afterwards, they were familiarized with the structure of if-then plans, identified a personal obstacle for adhering to social distancing guidelines, and a behavior that would help them to deal with it. Participants typed the obstacle, the behavior, and the resulting if (obstacle) – then (behavior) plan into text boxes. Pasting text into text boxes was disabled. Some examples of the if-then plans participants specified are: “If someone gets too close to me, I will move away from them”, “If I want to see my family, then I will have a video chat with them”, and “If I need to go to the store and the store is overly crowded, then I will leave and go elsewhere”. Participants formed and typed one if-then plan during the study but were encouraged to make additional if-then plans on their own following the same procedure.

After the intervention, participants indicated their *commitment to the social distancing guidelines* on four items (sample item: “I’m strongly committed to adhering to the social distancing guidelines”; Klein et al., 2001). The answers were averaged into a single score. In the planning condition, we additionally assessed the *intention to use plans* with one item (“I will use if-then plans to adhere to the social distancing guidelines”). Finally, participants answered demographic questions (income, education, employment, gender, age). We also asked whether they had already been diagnosed with COVID-19 or were quarantined because of it.

#### Measurements at T2

At T2, we again assessed the adherence to social distancing measures and the difficulty of adhering to social distancing measures analogously to T1. We assessed difficulty again in T2 because concurrent difficulty should be an important determinant of adherence and it seemed reasonable to assume that difficulty might have changed over the course of the week, given the rapid dynamics of the pandemic. Moreover, participants reported on their COVID-19 diagnosis and quarantine.

### Analytic Approach

We conducted an attrition analysis, comparing participants who dropped out after T1 to those who returned to T2, using independent t-tests and χ^2^-tests. We continued with a descriptive analysis of the main variables in our study before evaluating the effects of individual differences and the planning intervention.

#### Individual Differences in If-Then Planning, Self-Control, and Boredom

To adequately model the expected associations between individual differences in if-then planning, self-control, and boredom and adherence to social distancing guidelines in T1 and T2, we relied on structural equation modeling. Based on Wolff et al. (2020), we specified a model in which T1 difficulty *mediates* the associations between boredom with T1 and T2 adherence and *moderates* the associations of self-control with T1 and T2 adherence. As planning is a specific self-control strategy, we modeled it analogously to self-control. Figure 1 summarizes the proposed model. Besides these effects of interest, we accounted for the stability of behavior by including T1 difficulty and adherence as predictors of T2 difficulty and adherence, respectively. To account for potential effects of the intervention on T2 adherence, we included an intervention dummy variable (0 = control, 1 = planning). For examining indirect effects of boredom on adherence through difficulty, bias-corrected bootstrap confidence intervals based on 10,000 samples were computed. We report the model’s χ^2^-statistic along with the root mean square error of approximation (RMSEA), the standardized root mean square residual (SRMR), the comparative fit index (CFI), and the Tucker-Lewis index (TLI) to assess model fit. All continuous variables were mean-centered to enhance interpretability and remove nonessential multi-collinearity (Dalal & Zickar, 2012). Data from participants who dropped out after T1 were utilized by relying on full information maximum likelihood (FIML; e.g., Enders & Bandalos, 2001). All data and the code used for the analyses is available on OSF (https://osf.io/y2rdk).

#### If-Then Planning Intervention

We first conducted an *intention-to-treat analysis* with data from all participants to investigate the effects of assigning participants to a planning vs. control intervention. Because if-then planning interventions should only work when participants intend to use the strategy (Gollwitzer et al., 2010; Gollwitzer, 2014), we complemented this with a *per-protocol analysis*, focusing on the effect of actually receiving the intervention as intended. To this end, we identified participants in the if-then planning condition who intended to use plans (“intenders”; scoring 4 or 5 on the intention-to-use-plans item) in contrast to participants who expressed no such intention or were indifferent (“non-intenders”; scoring 1, 2, or 3). A majority of participants (*N* = 214, 75.6%) were classified as intenders. This combination of an intention-to-treat and a per-protocol analysis allows to simultaneously gauge the treatment effect of the if-then planning intervention and its potential to affect adherence given sufficient levels of compliance.

Because intenders and non-intenders were not randomly assigned, we checked for various characteristics on which they might have differed. We subjected commitment to an ANOVA with condition as single factor, aiming to rule out that differences in adherence merely reflect differences in commitment (e.g., an increased commitment after making if-then plans or among intenders). We proceeded with examining whether the planning intervention affected T2 adherence and difficulty by regressing them on condition dummies. We adjusted the analyses for commitment, T1 adherence, T1 difficulty, T2 difficulty, and demographics to account for potential confounds. We conducted analogous analyses for T2 difficulty. In all regressions, we used robust standard errors to hedge against potential heteroscedasticity.

## Results

### Attrition Analysis

Attrition rates were similar in the control and the planning condition, χ^2^(1) = 0.55, *p* = .459, also when distinguishing intenders and non-intenders, χ^2^(2) = 0.64, *p* = .726. Participants who dropped out of the study did not differ from the remaining participants in terms of demographics, *p*s > .08. However, they were less strongly committed to the guidelines (*M* = 3.60, *SD* = 0.99 vs. *M* = 4.29, *SD* = 0.90), *t*(572) = 7.36, *p* < .001, *d* = 0.75, found it more difficult to follow them (*M* = 3.19, *SD* = 1.14 vs. *M* = 2.45, *SD* = 1.15), *t*(572) = 6.33, *p* < .001, *d* = 0.64, and adhered to them less (*M* = 4.04, *SD* = 0.96 vs. *M* = 4.57, *SD* = 0.75), *t*(572) = 6.44, *p* < .001, *d* = 0.65. They scored higher on trait boredom (*M* = 3.18, *SD* = 1.06 vs. *M* = 2.51, *SD* = 1.07), *t*(572) = 6.13, *p* < .001, *d* = 0.62, and lower on self-control (*M* = 3.32, *SD* = 0.56 vs. *M* = 3.56, *SD* = 0.75), *t*(572) = 3.30, *p* = .001, *d* = 0.34, but displayed similar levels of planning (*M* = 3.96, *SD* = 0.58 vs. *M* = 3.88, *SD* = 0.63), *t*(572) = 1.26, *p* = .210, *d* = 0.13. In the planning condition, no attrition effect on the intention to use if-then plans emerged (*M* = 4.23, *SD* = 0.85 vs. *M* = 4.17, *SD* = 1.03), *t*(281) = 0.41, *p* = .686, *d* = 0.06.

### Descriptive Analysis

Descriptive statistics of the main variables are presented in Table 1. Adherence was high at T1 (overall *M* = 4.45, *SD* = 0.83) and decreased slightly among participants who returned to T2 (from *M* = 4.57, *SD* = 0.75 to *M* = 4.51, *SD* = 0.79), *t*(450) = 1.69, *p* = .093, *d* = 0.07. The difficulty scale (α at T1 = .89, α at T2 = .90) revealed that participants somewhat struggled with adherence at T1 (overall *M* = 2.61, *SD* = 1.19) and this did not change among participants returning to T2 (from *M* = 2.45, *SD* = 1.15 to *M* = 2.46, *SD* = 1.17), *t*(450) = 0.05, *p* = .959, *d* < 0.01. At concurrent and consecutive time points, adherence and difficulty were negatively correlated, *r* from −.39 to −.37. There were substantial correlations between adherence at T1 and T2, *r* = .65, and between difficulty at T1 and T2, *r* = .80, pointing to the stability of these constructs. Finally, participants with higher commitment (α = .79) adhered more to the guidelines at T1 and T2, *r* = .64 and *r* = .60, and found it less difficult, *r* = −.63 and *r* = −.59.

**Table 1:** *Means, Standard Deviations, and Correlations Between Key Variables.*

Variable	*M*	*SD*	α	1	2	3	4	5	6	7	8	9
1. T1 Adherence	4.45	0.83	―									
2. T2 Adherence	4.51	0.79	―	.65^***^								
				[.60, .70]								
3. T1 Difficulty	2.61	1.19	.89	−.38^***^	−.39^***^							
				[−.45, −.31]	[−.46, −.31]							
4. T2 Difficulty	2.45	1.17	.90	−.37^***^	−.39^***^	.80^***^						
				[−.45, −.29]	[−.47, −.31]	[.76, .83]						
5. T1 Diagnosis /	0.10	0.30	―	−.23^***^	−.18^***^	.30^***^	.30^***^					
Quarantine				[−.30, −.15]	[−.27, −.09]	[.22, .37]	[.21, .38]					
6. T2 Diagnosis /	0.07	0.26	―	−.18^***^	−.21^***^	.31^***^	.30^***^	.65^***^				
Quarantine				[−.27, −.09]	[−.30, −.12]	[.22, .39]	[.22, .39]	[.60, .70]				
7. Commitment	4.14	0.96	.83	.64^***^	.60^***^	−.63^***^	−.59^***^	−.30^***^	−.30^***^			
				[.59, .69]	[.54, .66]	[−.68, −.58]	[−.64, −.52]	[−.37, −.22]	[−.38, −.22]			
8. Trait Boredom	2.65	1.10	.93	−.29^***^	−.27^***^	.65^***^	.59^***^	.32^***^	.30^***^	−.53^***^		
(SBPS)				[−.37, −.22]	[−.35, −.18]	[.60, .70]	[.52, .64]	[.24, .39]	[.21, .38]	[−.59, −.47]		
9. Trait Self-Control	3.51	0.72	.91	.34^***^	.28^***^	−.42^***^	−.38^***^	−.18^***^	−.16^***^	.41^***^	−.70^***^	
(CFSCS)				[.27, .41]	[.19, .36]	[−.49, −.35]	[−.46, −.30]	[−.26, −.10]	[−.25, −.07]	[.34, .48]	[−.74, −.65]	
10. If-then Planning	3.89	0.62	.79	.29^***^	.19^***^	.09^*^	.05	−.01	.04	.12^***^	−.09^*^	.38^***^
(ITPS)				[.21, .36]	[.10, .28]	[.01, .17]	[−.05, .14]	[−.10, .07]	[−.05, .14]	[.04, .20]	[−.17, −.01]	[.31, .45]

*Note.* Values in square brackets indicate the 95% confidence intervals. Statistics involving T2 adherence or T2 difficulty are based on *N* = 451 observations, all other statistics are based on *N* = 574 observations

^*^ *p* < .05. ^***^ *p* < .001

**Fig. 2:**
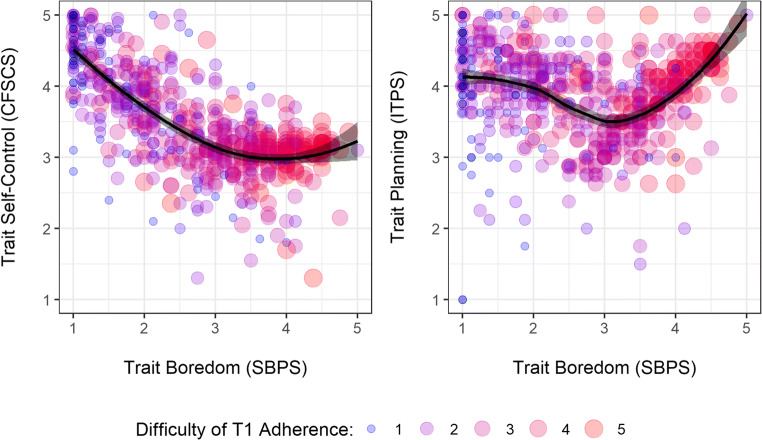
*Relationship Between Trait Measures of If-Then Planning, Self-Control, and Boredom and the Difficulty to Adhere to Social Distancing Guidelines at T1.* The solid line represents a non-parametric Loess curve fitted locally to the data along with its 95% confidence interval displayed as shaded gray region

The measures of trait boredom (SBPS; α = .93), self-control (CFSCS; α = .91) and planning (ITPS; α = .79) showed good to excellent internal consistency. Boredom and self-control were negatively correlated, *r* = −.70, and both correlated with adherence and its difficulty at T1 and T2, |*r*| from .27 to .65. Planning was also correlated with adherence at T1 and T2, *r* = .29 and *r* = .19, but its correlations with difficulty were small, *r* = .09 and *r* = .05. Moreover, planning was correlated with self-control, *r* = .38, but only weakly with boredom, *r* = −.09. Figure 2 sheds light on this: The association between boredom and if-then planning was quadratic, with high values of planning among participants with low or high boredom. For participants with high boredom, this means that their low levels of self-control (left panel) were matched by a high level in planning (right panel). This might explain why they found it difficult to adhere to the guidelines (low self-control) and yet displayed encouragingly high levels of adherence (high planning).

### Individual Differences in If-Then Planning, Self-Control, and Boredom as Predictors of Adherence

For an overview of the proposed model, see Table 2. Model fit was excellent and the results were robust to adjusting for demographics. We adjusted for potential differences between the control and the intervention condition in terms of T2 difficulty and adherence by including a corresponding dummy variable.

**Table 2:** *Results of the Structural Equation Model for Predicting T2 Adherence*

	Without Demographics			With Demographics		
	*b*	β	*SE*	*b*	β	*SE*
**T1 Adherence on**						
T1 Difficulty	−0.302^***^	−0.433	0.034	−0.299^***^	−0.430	0.035
Trait Boredom	0.069	0.092	0.051	0.065	0.087	0.049
Trait Self-Control	0.189^*^	0.164	0.075	0.203^**^	0.177	0.074
Trait Self-Control X T1 Difficulty	0.105^*^	0.106	0.045	0.110^**^	0.111	0.042
Trait Planning	0.455^***^	0.339	0.073	0.452^***^	0.340	0.070
Trait Planning X T1 Difficulty	0.156^*^	0.142	0.070	0.167^**^	0.153	0.064
R^2^	0.293					
**T1 Difficulty on**						
Trait Boredom	0.702^***^	0.652	0.031	0.715^***^	0.664	0.033
R^2^	0.425					
**T2 Adherence on**						
T2 Difficulty	−0.086^*^	−0.126	0.042	−0.080^†^	−0.117	0.042
T1 Adherence	0.563^***^	0.571	0.082	0.556^***^	0.562	0.083
T1 Difficulty	−0.092^†^	−0.133	0.049	−0.112^*^	−0.163	0.051
Intervention	0.071	0.044	0.055	0.073	0.045	0.056
SBPS	0.049	0.067	0.041	0.044	0.059	0.044
CFSCS	0.018	0.015	0.060	0.003	0.002	0.061
CFSCS x T1 Difficulty	−0.020	−0.020	0.040	−0.026	−0.027	0.042
ITPS	0.138^*^	0.104	0.061	0.161^*^	0.122	0.062
ITPS x T1 Difficulty	0.090^†^	0.083	0.049	0.115^†^	0.106	0.051
R^2^	0.507					
**T2 Difficulty on**						
T1 Adherence	−0.089^†^	−0.061	0.047	−0.083^†^	0.194	0.047
T1 Difficulty	0.682^***^	0.677	0.045	0.676^***^	−0.057	0.045
Intervention	0.050	0.021	0.064	0.036	0.671	0.064
SBPS	0.182^***^	0.167	0.045	0.211^***^	0.015	0.047
R^2^	0.675					
**Fit statistics**	**χ**^**2**^**(8)**	***p***	**RMSEA**	**χ**^**2**^**(8)**	***p***	**RMSEA**
	11.28	.186	0.027	10.27	.247	0.022
	**CFI**	**TLI**	**SRMR**	**CFI**	**TLI**	**SRMR**
	0.996	0.987	0.020	0.998	0.980	0.007

*Note:* SBPS = Short Boredom Proneness Scale; CFSCS = Capacity for Self-Control Scale; ITPS = If-Then Planning Scale; RMSEA = Root Mean Square Error of Approximation; CFI = Comparative Fit Index; TLI = Tucker-Lewis Index; SRMR = Standardized Root Mean Square Residual. We report unstandardized (*b*) and standardized coefficients (β) with robust standard errors (*SE*). Missing data was dealt with using full-information maximum likelihood (FIML)

^†^*p* < .1. ^*^p < .05. ^**^p < .01. ^***^p < .001

#### Adherence and Difficulty at T1

T1 adherence was associated with T1 difficulty, *b* = −0.30, β = −0.43, *SE* = 0.03, *p* < .001. More importantly, higher trait boredom was associated with higher T1 difficulty, *b* = 0.70, β = 0.65, *SE* = 0.03, *p* < .001, but not with T1 adherence, *b* = 0.07, β = 0.09, *SE* = 0.09, *p* = .172. The indirect negative effect of boredom on T1 adherence via T1 difficulty was significant, *b* = −0.21, 95% CI [−0.26, −0.16], β = −0.28, 95% CI [−0.35, −0.22], *SE* = 0.03, *p* < .001, as was the total effect, *b* = −0.14, β = −0.19, *SE* = 0.05, *p* = .002. Higher self-control was associated with higher T1 adherence, *b* = 0.19, β = 0.16, *SE* = 0.05, *p* = .012, especially when T1 difficulty was higher, *b* = 0.11, β = 0.11, *SE* = 0.05, *p* = .019. Analogously, higher if-then planning was associated with higher T1 adherence, *b* = 0.46, β = 0.34, *SE* = 0.07, *p* < .001, especially when T1 difficulty was higher, *b* = 0.16, β = 0.14, *SE* = 0.07, *p* = .025. These results replicate findings by Wolff et al. (2020) and complement them by showing that if-then planning as a specific self-control strategy has similar and even stronger associations with adherence than self-control.

#### Adherence and Difficulty at T2

T2 adherence was associated with T1 adherence, *b* = 0.56, β = 0.57, *SE* = 0.08, *p* < .001, as well as with T2 difficulty, *b* = −0.09, β = −0.13, *SE* = 0.04, *p* = .040. Higher trait planning was associated with higher T2 adherence, *b* = 0.14, β = 0.10, *SE* = 0.06, *p* = .023, and this association tended to be stronger with increasing T1 difficulty, *b* = 0.09, β = 0.08, *SE* = 0.05, *p* = .064. Neither boredom nor self-control were associated with T2 adherence, *p* > .23, but higher boredom was associated with higher T2 difficulty, *b* = 0.18, β = 0.17, *SE* = 0.05, *p* < .001, beyond T1 difficulty, *b* = 0.68, β = 0.68, *SE* = 0.05, *p* < .001.

### Effects of the If-Then Planning Intervention on Adherence

No difference between the control and the planning condition emerged in terms of their commitment to the social distancing guidelines, *F*(1, 572) = 0.17, *p* = .683, ${\upeta}_{\mathrm{g}}^2$ < .001. Further distinguishing between intenders and non-intenders in the planning condition revealed a significant effect, *F*(2, 571) = 8.49, *p* < .001, ${\upeta}_{\mathrm{g}}^2$ = .029. Commitment was lower in the planning (non-intender) condition (*M* = 3.75, *SD* = 1.09) compared to the control (*M* = 4.13, *SD* = 0.98), *t*(571) = 2.95, *p* = .010, *d* = .40, and the planning (intenders) condition (*M* = 4.29, *SD* = 0.86), *t*(571) = 4.10, *p* < .001, *d* = .57, while the difference between control and planning (intenders) condition was not significant, *t*(571) = 1.92, *p* = .166, *d* = .17. Differences between the control and the planning (intenders) condition in T2 adherence or difficulty are thus unlikely to reflect differences in commitment. Still, we adjusted for commitment when differentiating between intenders and non-intenders to account for the existing variance and to obtain the most precise estimate of the intervention effect.

#### Intention-to-Treat Analysis

We first regressed T2 adherence on condition (see Table 3), which revealed no significant difference, *b* = 0.06, β = 0.08, *SE* = 0.07, *p* = .419 (Model 1). In terms of Cohen’s *d*, the difference between conditions was very small (*d* = .12; control intervention: *M* = 4.48, *SD* = 0.82; if-then planning intervention: *M* = 4.54, *SD* = 0.75). Also, we observed no significant interaction between condition and if-then planning, self-control, or boredom, *p*s ≥ .13 (Model 2). This suggests that the if-then planning intervention was not effective in increasing adherence to social distancing guidelines.

**Table 3:** *Regression of T2 Adherence on Condition and Control Variables*

**Variable**	**Intention-to-Treat Analysis**		**Per-Protocol Analysis**			
	**Model 1**	**Model 2**	**Model 3**	**Model 4**	**Model 5**	**Model 6**
Intercept	4.48 (0.05)^***^	1.77 (0.65) ^**^	4.48 (0.05)^***^	2.27 (0.20)^***^	1.29 (0.38)^***^	0.95 (0.49)
Condition	0.06 (0.07)	−1.08 (0.85)				
Commitment		0.53 (0.07) ^***^		0.52 (0.04)^***^	0.26 (0.06)^***^	0.27 (0.06)^***^
Condition × Commitment		0.03 (0.10)				
Self-control		0.01 (0.07)				
Condition × Self-Control		0.16 (0.10)				
Boredom		0.02 (0.07)				
Condition × Boredom		0.12 (0.09)				
If-then Planning		0.09 (0.09)				
Condition × If-Then Planning		0.04 (0.11)				
Condition (intenders)			0.18 (0.07)^*^	0.12 (0.06)^*^	0.10 (0.05)^*^	0.11 (0.05)^*^
Condition (non-intenders)			−0.31 (0.15)^*^	−0.09 (0.12)	−0.05 (0.10)	−0.05 (0.10)
T2 Difficulty					−0.05 (0.04)	−0.05 (0.04)
T1 Adherence					0.46 (0.08)^***^	0.46 (0.09)^***^
T1 Difficulty					0.02 (0.04)	−0.01 (0.05)
Demographics						included
Num. obs.	451	451	451	451	451	451
R^2^	0.00	0.38	0.04	0.37	0.46	0.51
Adj. R^2^	−0.00	0.37	0.03	0.37	0.45	0.48
L.R.	0.65	216.63	17.08	211.00	278.21	326.25

*Note.* We report unstandardized coefficients with robust standard errors in parentheses. “Condition” refers to a dummy variable coded as 0 for the control condition and as 1 for the planning condition (or intenders and non-intenders). The demographic variables comprised age, gender, education, employment status, and income. ^*^p < .05. ^**^p < .01. ^***^p < .001

#### Per-Protocol Analysis

Intenders in the planning condition showed more adherence than control participants, *b* = 0.18, β = 0.23, *SE* = 0.07, *p* = .010, while non-intenders adhered less than control participants, *b* = −0.31, β = −0.38, *SE* = 0.15, *p* = .043 (Table 3, Model 3). However, this is an upper bound of the potential intervention effect because intenders and non-intenders systematically differed from control participants (e.g., commitment). To arrive at a more genuine estimate of the potential intervention effect (i.e., adjusted for potential confounds), it is paramount to account for these characteristics.

Indeed, after adjusting for commitment non-intenders no longer differed from control participants, *b* = −0.09, β = −0.12, *SE* = 0.12, *p* = .433 (Model 4), while intenders still adhered more to the guidelines, *b* = 0.12, β = 0.16, *SE* = 0.06, *p* = .028. This suggests that the higher adherence at T2 among intenders compared to control participants is not driven by higher commitment. Similarly, adjusting for T1 adherence, T1 difficulty, and T2 difficulty (Model 5) and for demographics (Model 6) did not change these results. In terms of Cohen’s *d*, the effect was small-to-medium (.20 to .37) across models. Taken together, the planning intervention helped participants who intended to use plans. Inspection of the data showed that they maintained similar levels of adherence at T2 compared to T1 (*M* = 4.73 to *M* = 4.70), whereas adherence decreased among other participants (control: *M* = 4.66 to *M* = 4.55, non-intenders: *M* = 4.53 to *M* = 4.45). Thus, the intervention did not *increase* adherence but was essential to *maintain* it. Analogous analyses for T2 difficulty revealed no differences between conditions, *p* > .14, suggesting that the intervention did not affect difficulty.

## Discussion

At the time of writing many countries have eased some of the most restrictive COVID-19 containment measures. While easing the restrictions might reduce the experience of boredom, substantial self-control demands remain in place for the individual. Indeed, several of the remaining social distancing guidelines—such as keeping a given distance at all time or avoiding gatherings—require self-control. These measures slow the spread of the virus (Gollwitzer et al., 2020) and might be necessary for a prolonged time (Kissler et al., 2020). Moreover, the current easing of restrictions follows a period that might have been experienced as effortful, thus individuals might not be willing to further exert effort. Therefore, it is paramount to understand the psychological processes behind the adherence to social distancing guidelines and the difficulties associated with it. Moreover, interventions that might help individuals to deal with difficulties associated with social distancing guidelines are urgently needed. Here, we replicate and extend first evidence for the role of boredom and self-control in the COVID-19 response (Martarelli & Wolff, 2020; Wolff et al., 2020) by highlighting the crucial role that making if-then plans can play in the current situation.

### The Role of Individual Differences in If-Then Planning, Self-Control, and Boredom in Explaining Adherence to Social Distancing Guidelines

First, we found empirical support for associations of individual differences in if-then planning, self-control, and boredom with the adherence to social distancing guidelines and its difficulty at T1. Higher trait boredom was associated with higher difficulty, which mediated the effect of boredom on adherence. The indirect effect of boredom on adherence revealed that difficulty is an important mediator and thus an integral part of the mechanism that underlies adherence to social distancing guidelines. Recent theorizing ascribes a functional role to the experience of boredom in terms of preparing a person to seek out more rewarding alternatives (e.g., Bench & Lench, 2013; Elpidorou, 2021) thereby making sustained adherence to social distancing guidelines more difficult. Further, high trait self-control as well as trait if-then planning were directly associated with higher adherence to social distancing guidelines, especially among individuals who perceived adherence as difficult. This finding highlights the key roles of self-control and if-then planning for enhancing adherence to the guidelines and fostering adaptive behavior.

When considering the impact of individual differences in boredom, self-control, and if-then planning on adherence and difficulty measured at T2, many findings were robust. Boredom again emerged as a predictor of difficulty, which mediated its adverse effect on adherence. However, if-then planning played a more important role when it came to maintaining adherence to the guidelines than having self-control per se. According to recent functional theorizing on self-control, the sensation that accompanies the application of self-control signals that one should avoid investing further effort (Wolff & Martarelli, 2020). We propose that if-then planning makes adherence less effortful and thus maintains the willingness to further apply effort (Freydefont et al., 2016; Wolff et al., 2018). In other words, stronger inclinations to engage in if-then planning prepare individuals to deal more efficiently with the various demands associated with adhering to social distancing guidelines. A similar line of reasoning emerges from research showing that people high in self-control are less likely to be confronted with temptations (Hofmann et al., 2012) because they avoid tempting situations (Ent et al., 2015). More generally, they display rather adaptive habits (e.g., de Ridder et al., 2012), which has been linked to a greater propensity to make if-then plans and thereby automate one’s behavior (Gillebaart & de Ridder, 2015). Accordingly, if-then planning might be a mechanism that makes self-controlled behavior less effortful and more successful.

### The Effects of an If-Then Planning Intervention on Adherence to Social Distancing Guidelines

Second, we tested the effect of prompting people to make if-then plans on adherence to social distancing guidelines. While making if-then plans had no treatment effect as indicated by the intention-to-treat analysis, the per-protocol analysis indicated that if-then planning maintained high levels of adherence among individuals intending to use the plans while having no effect on individuals without such an intention. The estimated effect on T2 adherence was small-to-medium (Cohen’s *d* between .20 and .37) and extremely robust when adjusting for various control variables. This is remarkable for an intervention that is associated with literally no costs and that can be easily distributed. Yet, about 25% of the participants had little intention to use if-then plans in the first place, thwarting the overall effectiveness of the intervention. Given that adherence must be the highest level possible for social distancing measures to be effective, even few cases of non-adherence jeopardize the efforts to contain the pandemic. Thus, interventions based on if-then planning should be further examined as a simple and effective way to facilitate adherence to the social distancing guidelines in the general public. Finally, one might argue that people could not possibly adhere even more to the guidelines than at T1. However, we found *decreasing* adherence in the control condition over the course of only one week, suggesting that people struggle with maintaining adherence unless they were instructed to make if-then plans.

### Limitations and Future Directions

Some limitations of the study should be considered. First, the attrition analysis comparing participants who dropped out after T1 to those who returned at T2 suggests that individuals who struggle most with the social distancing guidelines can be difficult to target with an intervention. This was also reflected in the lack of an intervention effect among participants who returned to T2 but displayed little commitment to the social distancing guidelines and had little intention to use the if-then plans. This is not a specific limitation of the intervention we used, as many responses to COVID-19 require compliance (including pharmaceutical measures like vaccines). Future research would nevertheless benefit from investigating complementary interventions that focus on increasing compliance, such as enhancing the personal meaning of social distancing guidelines (Brooks et al., 2020).

Second, it is important to mention that all participants in the if-then planning intervention condition had to make an if-then plan to complete the study. Hence, even non-intenders learned about the intervention but showed no higher adherence at T2 than participants in the control condition. This provides further arguments for a focus on establishing compliance with the if-then planning intervention in future research: without such compliance, leading people through a planning procedure seems to be ineffective.

Third, it should be considered that the delay between T1 and T2 was of only one week. Accordingly, the effect we observed in the analysis of personality characteristics and in the per-protocol analysis reflect rather short-term changes that we could not check for their stability over longer periods of time. This is partly due to the nature of a pandemic, which makes it difficult to observe behaviors over long time horizons (e.g., because social distancing guidelines and incidence rates are highly dynamic). Still, it would be important to focus on predicting long-term changes of behavior in future research.

A fourth limitation of our study pertains to the characteristics of our sample and our methodological approach. We obtained self-reports from a sample of US adults that was recruited online via a paid crowd-sourcing platform and in which male participants were overrepresented (64%). While an online study with paid participants was the only way to obtain a sufficiently large sample during the pandemic, it might limit our ability to investigate behavior change (e.g., due to rather low incentives). With regard to our focus on the US, it should be noted that the current situation is evolving fast and there are differences between countries, both in terms of the spread of the virus and of the response to it. Importantly, the US had the highest number of COVID-19 cases at that time and validated measures for all trait constructs were readily available in English. Regarding the measures, self-reported adherence to social distancing guidelines has been investigated in previous research (Wolff et al., 2020) and validated with behavioral data (for instance, using activity tracking data from mobile phones; Gollwitzer et al., 2020). Our results also converge with research on adherence to social distancing guidelines using alternative measures (Boylan et al., 2021), speaking to the robustness of our approach.

Finally, it is also worth highlighting that we replicated findings of Wolff et al. (2020) in showing that individual differences in boredom and self-control are associated with adherence to social distancing. However, more research is needed to assess the generalizability and robustness of our findings. For instance, we assumed that participants with higher trait boredom experience boredom more frequently and more intensely than participants low in trait boredom. While this assumption has been supported in recent research (Tam et al., 2021), we did not explicitly test it by including a measure of state boredom. It seems to be a fruitful approach in future research to examine whether state boredom mediates the effects of trait boredom on adherence (and behavior in general). Moreover, we based our hypotheses on research suggesting that self-control moderates the effects of boredom on adherence (Wolff et al., 2020). Interestingly, the findings of this research have been replicated based on a model in which boredom mediated the relationship between self-control and adherence (Boylan et al., 2021). Obviously, in correlational designs, different conceptualizations of the mechanisms by which boredom and self-control affect behavior lead to similar conclusions. We did not set up our study to pitch these two conceptualizations against each other. Therefore, future research should disentangle the interplay between boredom and self-control more closely with experimental approaches (e.g., Bieleke et al., 2021). A final interesting approach would be to complement our measure of the perceived difficulty of adherence with a measure of self-efficacy as a well-established social cognitive concept (Bandura, 1997). Self-efficacy pertains to the subjective expectation of being able to deal with new or difficult situations on the basis of one’s own competencies. As such, it is conceivable that self-efficacy moderates the relationship between perceived difficulty of adhering to social distancing guidelines and actual adherence.

## Conclusion

In conclusion, the present research provides an important contribution by revealing the role of boredom, self-control, and if-then planning in explaining adherence to social distancing guidelines. Taking into account individual differences in these constructs can help in tailoring specific interventions adapted to individual personalities and situations. Moreover, we tested a simple if-then planning intervention to increase adherence. Our data suggest that this intervention is promising, as it maintained high levels of adherence among people who intended to use it. However, lack of compliance with the intervention thwarted its general effectiveness, calling for continued research on how to improve the design of if-then planning interventions (e.g., by raising compliance).

### Data Availability Statement

The datasets generated and analyzed in the current study are available in the OSF repository, https://osf.io/y2rdk/
